# The Collaborative Outcome Study on Health and Functioning during Infection Times (COH-FIT): Results from Cyprus

**DOI:** 10.3390/jcm13185395

**Published:** 2024-09-12

**Authors:** Evangelia Papatriantafyllou, Dimitris Efthymiou, Kyriakos Felekkis, Marco Solmi, Christoph U. Correll, Trevor Thompson, Andrés Estradé, Sofia Tsokani, Katerina-Maria Kontouli, Georgios Seitidis, Ourania Koutsiouroumpa, Dimitris Mavridis, Christos Christogiannis, Emilia Vassilopoulou

**Affiliations:** 1 Department of Nutrition and Dietetics, School of Health Sciences, International Hellenic University, 57400 Thessaloniki, Greece; e.papatriantafyllou@hotmail.com; 2 Nous Thrapy Center, 54621 Thessaloniki, Greece; dimitrisefthy@gmail.com; 3 Department of Life Sciences, School of Life and Health Sciences, Universiy of Nicosia, Nicosia 2417, Cyprus; felekkis.k@unic.ac.cy; 4 Department of Psychiatry, Faculty of Medicine, University of Ottawa, Ottawa, ON K1N 6N5, Canada; marco.solmi83@gmail.com; 5 Department of Mental Health, The Ottawa Hospital, Ottawa, ON K1H 8L6, Canada; 6 Clinical Epidemiology Program, Ottawa Hospital Research Institute (OHRI), Ottawa, ON K1Z 7K4, Canada; 7 Department of Child and Adolescent Psychiatry, Charité Universitätsmedizin Berlin, 10117 Berlin, Germany; ccorrell@northwell.edu; 8 Center for Psychiatric Neuroscience, The Feinstein Institute for Medical Research, Manhasset, NY 11030, USA; 9 Department of Psychiatry, Northwell Health, Zucker Hillside Hospital, Glen Oaks, NY 11004, USA; 10 Department of Psychiatry and Molecular Medicine, Donald and Barbara Zucker School of Medicine at Hofstra/Northwell, Hempstead, NY 11549, USA; 11 German Center for Mental Health (DZPG), Partner Site Berlin, 10117 Berlin, Germany; 12 Centre for Chronic Illness and Ageing, University of Greenwich, London SE10 9LS, UK; t.thompson@greenwich.ac.uk; 13 Early Psychosis: Interventions and Clinical-Detection (EPIC) Lab, Department of Psychosis Studies, Institute of Psychiatry, Psychology & Neuroscience, King’s College London, London SE5 8AB, UK; andres.estrade_vaz@kcl.ac.uk; 14 Department of Primary Education, School of Education, University of Ioannina, 45110 Ioannina, Greece; sofia.tsokani@gmail.com (S.T.); kmkontouli@uoi.gr (K.-M.K.); o.koutsiouroumpa@uoi.gr (O.K.); dmavridi@uoi.gr (D.M.); c.christogiannis@uoi.gr (C.C.); 15 Department of Psychology, School of Social Sciences, University of Ioannina, 45110 Ioannina, Greece; g.seitidis@uoi.gr

**Keywords:** COVID-19 pandemic, mental health, well-being, Cyprus, global health crisis

## Abstract

Many studies have shown that COVID-19 caused many problems in mental health. This paper presents the results of the Cyprus sample, part of the global initiative named “The Collaborative Outcomes Study on Health and Functioning during Infection Times” (COH-FIT). **Methods:** The study took place from April 2019 to January 2022, using the Greek version of the online standard COH-FIT questionnaire on 917 Cypriot adults. Weighted *t*-tests were applied to test the differences between pre-pandemic and intra-pandemic scores using the anesrake package. **Results:** Participant responses indicated a significant negative impact of the pandemic on measures of mental health (−7.55; 95% CI: −9.01 to −6.07), with worsening in the scores for anxiety (12.05; 95% CI: 9.33 to 14.77), well-being (−11.06; 95% CI: −12.69 to −9.45) and depression (4.60; 95% CI: 2.06 to 7.14). Similar negative effects were observed for feelings of anger (12.92; 95% CI: 10.54 to 15.29), helplessness (9.66; 95% CI: 7.25 to 12.07), fear (22.25; 95% CI: 19.25 to 25.26), and loneliness (12.52; 95% CI: 9.94 to15.11). Increased use of social media (0.89; 95% CI: 0.71 to 1.09), internet (0.86; 95% CI: 0.67 to 1.04), and substance consumption (0.06; 95% CI: 0.00 to 0.11) were reported, along with a significant decrease in physical health (−3.45; 95% CI: −4.59 to −2.32), self-care (−7.10; 95% CI: −9.00 to −5.20), and social function (−11.27; 95% CI: −13.19 to −9.35), including support (−0.72; 95% CI: −1.09 to −0.34) and family function (−7.97; 95% CI: −9.90 to −6.05). **Conclusions:** The COVID-19 pandemic significantly affected the daily life and emotional well-being of Cypriots. Identifying factors that influence vulnerability and resilience is essential to prioritize mental health support and address the long-term effects of the pandemic.

## 1. Introduction

The emergence of Coronavirus disease 2019 (COVID-19), caused by the SARS-CoV-2 virus, evolved rapidly into a global pandemic of unprecedented scale [1,2,3]. To mitigate the transmission of the virus, stringent measures were introduced across diverse regions of the world to restrict transmission of the virus and safeguard public health [4]. The COVID-19 pandemic itself and the measures instituted to control its spread had profound adverse effects on the physical and mental well-being of individuals, resulting in a deterioration of the overall quality of life of the general population [4,5,6]. Globally, the COVID-19 pandemic exerted a substantial negative impact on mental health. The experience of uncertainty, social distancing, and disruption of daily life routines contributed to the exacerbation of mental health issues, as documented by studies that indicate an increase in stress, anxiety, depression, fear, loneliness, and social isolation [7,8,9].

The pandemic-related measures and social distancing guidelines led to changes in lifestyle, with a significant reduction in physical activity, an increase in body weight [10,11,12], and limited opportunities for social contact [12]. Weight trends during the COVID-19 pandemic exhibited variability, with some individuals experiencing weight gain following disruption of their routine, reduced activity, and changes in eating habits, while others directed their attention during isolation towards fitness and healthy eating [10,11,12]. Studies focused on addictive behavior during the pandemic [13,14,15] revealed variations in patterns of alcohol and substance use, with a substantial proportion of people reporting increased intake in response to the stress of the new circumstances, while others abstained for reasons of health or personal growth. Survey data have revealed a wide range in the prevalence of alcohol consumption in the general population, from 21.7% to 72.9%, and a rise in the use of other substances, with a reported range of from 3.6% to 17.5% [16].

In addition, the COVID-19 pandemic was associated with a significant surge in internet use, driven by the introduction of remote work and online education and by the need for people for virtual social interaction [17]. Furthermore, gaming emerged as a prominent form of home entertainment alongside other, potentially more harmful, online activities, including gambling and pornography [17,18]. 

The Collaborative Outcomes Study on Health and Functioning during Infection Times (COH-FIT) is a global initiative conducted across 156 countries, designed to evaluate comprehensively the impact of COVID-19 on health and well-being, encompassing physical and mental health, social outcomes, and healthcare functioning [4,19]. The study data were collected through an online anonymous questionnaire, which was translated into 30 languages to ensure widespread participation. The study took into consideration the diverse effects observed among different population groups, influenced by both non-modifiable and modifiable risk factors.

Cyprus reported its first COVID-19 cases on 9 March 2020. Relevant interventions, commonly known as “lockdown” measures, were initiated promptly, including school closure (10 March 2020), the shutdown of public spaces (13 March 2020), and stay-at-home orders (24 March 2020). These mandatory measures, along with general recommendations, were designed to promote staying at home and working from home when possible, the practice of physical distancing in the workplace and in social settings, and the implementation of specific workplace safety measures [20].

The objective of this present study was to present selected data from the study conducted in Cyprus as part of the COH-FIT research project in order, firstly, to evaluate the factors influencing the physical and mental health of adults in Cyprus during the COVID-19 pandemic and, secondly, to identify factors associated with either increased susceptibility or resilience to the adverse physical and mental health challenges posed by the pandemic and the resultant varying levels of restrictions imposed during COVID-19 pandemic. Ultimately, this project aimed to identify both risk factors and protective factors in the Cypriot population that could provide the basis for the development of prevention and intervention programs tailored specifically for the unique challenges posed by the COVID-19 pandemic. 

## 2. Materials and Methods

### 2.1. Participants

The Cyprus COH-FIT survey took place between April 2019 and January 2022, using an anonymous online Greek version of the COH-FIT questionnaire, which was distributed through various channels, including popular social media platforms such as Facebook, Instagram, and LinkedIn, and through email and telephone outreach. Respondents who were aged over 18 years and chose to take part in the study were required to provide their written informed consent for participation upon accessing the online questionnaire platform. This study was conducted in accordance with the principles of the Declaration of Helsinki, and ethical approval was granted by the Cypriot Ethics Committee (ΕΕΒΚ ΕΠ 2020 29).

### 2.2. The COH-FIT Questionnaire

The COH-FIT questionnaire is available on the official survey page [21] (https://www.coh-fit.com/, accessed on 17 October 2023), and it has been comprehensively described and validated by Solmi and colleagues [4,19,22]. It was designed to provide insight into the challenges that individuals faced during this global health crisis. The information was gathered voluntarily and anonymously to protect the privacy of participants. The initial section of the questionnaire collected demographic information, including age range, sex, ethnicity, occupation, household composition, height, and weight. 

Responses on most of the items in the questionnaire were requested for two different time points, i.e., ‘two weeks before the outbreak of the COVID-19 pandemic’ in Cyprus, named “pre-pandemic”, and ‘during the last two weeks prior to the survey response during the COVID-19 pandemic’, named “intra-pandemic”. 

As one co-primary outcome, well-being was measured with «The World Health Organization-Five Well-Being Index (WHO-5)» in the COH-FIT study [19,22]. In order to assess the mental health of the participants, we used the questionnaire items, which covered the measurement of anxiety, depressive symptoms, post-traumatic symptoms, obsessive-compulsive symptoms, bipolar symptoms, and psychotic symptoms, and the evaluation of stress, sleep problems, and concentration difficulties, using a reduced number of items from validated questions. All of these items together are called the “psychopathology extended score” (p-extended score). During the validation process, the validated p-score, as the second co-primary outcome in the COH-FIT study, included only mental health domains that showed correlations between the abbreviated questionnaires and the original, validated questionnaire of >0.50, leading to the exclusion of obsessive-compulsive symptoms and bipolar symptoms from the validated p-score [22]. In addition, items were included that elicited the changes in daily activities, physical activity, social contact, family life, and addictive behaviors. All the items are presented in a continuous visual analog scale format, ranging from 0 to 100, apart from some demographic information and a question about how the participant learned about the survey. All responses to the questionnaire were optional, and the participants were free to discontinue their participation at any point during the survey. 

Altogether, 1.248 respondents participated during the study period, but 311 were excluded because of a pre-ethics approval completion date and/or lack of consent. This left 937 respondents whose questionnaires were available for analysis, of which 20 were excluded as they were aged under 18 years, resulting in a final number of 917 adult COH-FIT Cypriot participants.

### 2.3. Statistical Analysis of Data

To ensure the representativeness of the Cypriot sample, the raking method was employed to weigh the data, aligning it with the characteristics of the actual Cypriot population according to sex, age, and region [23]. Using this approach, we aimed to mitigate bias in the point estimates by incorporating weights derived from the sample distribution of the actual Cypriot population. For the available data, we performed a Complete Case Analysis (CCA). This method provides unbiased estimates under the Missing Completely at Random (MCAR) mechanism, which means that the missingness of data in a variable is unrelated to the values of that variable. The missingness was quite large (e.g., more than 40%) in items that appeared towards the end of the questionnaire. This hindered any imputation method and is probably due to tiredness and lack of interest of the respondents, an assumption that is compatible with MCAR.

For quantitative variables, weighted mean values ± standard deviation (±SD) and quartiles were used. The statistical significance of the difference between “pre-pandemic” and “intra-pandemic” values was assessed using weighted *t*-tests (not taking time into consideration). Finally, the way in which the outcomes of interest varied according to the date of completion of the questionnaire (i.e., the time that had passed since the introduction of the measures) was presented graphically. In the graphs, the change from baseline (CFB) was regressed on time, and the corresponding confidence intervals (CI) and *p*-values were derived. The results of CFB refer to the monthly changes in results. The significance level was set at 5%. Also, we provide in the Supplementary Materials Figures S1–S11 with the weighted Pearson correlations for the variables that are included in the tables, to enhance the robustness of our results. 

We based inferences on the weighted sample, not the original (unweighted) sample. Analysis was conducted in R using the package anesrake (version 2022) [24].

## 3. Results

### 3.1. Demographic Characteristics

The COH-FIT Cypriot sample consisted of 917 participants, comprising 68.4% females, 31.0% males, 0.5% non-binary, and 16% with missing data. 

The majority of the participants self-identified as white (95.5% pre-weighting, 95.2% post-weighting) and reported that they were either married or in a cohabiting relationship (57.5% pre-weighting, 60.4% post-weighting). In terms of educational level, more than one-third (71.7% pre-weighting, 68.0% post-weighting) had a university or college education, and 20.4% pre-weighting or 21.9% post-weighting had completed their education on graduation from high school. The demographic characteristics of the study population are shown in Table S1 (Supplementary Materials). 

In the weighted sample, two-thirds of the participants reported being employed, indicating that a significant portion of the population remained actively engaged in work during the pandemic. The majority (87.7%) reported that they did not experience job loss in this challenging period. The unweighted and weighted employment status of the study population are shown in Table S2 (Supplementary Materials).

Most of those employed were full-time workers (65.8%), and they continued to attend at their workplaces as usual during the pandemic. A smaller percentage reported job loss (12.3%) during this period, and a few (16.0%) were able to work from home. 

### 3.2. Mental Health

**Figure 1:**
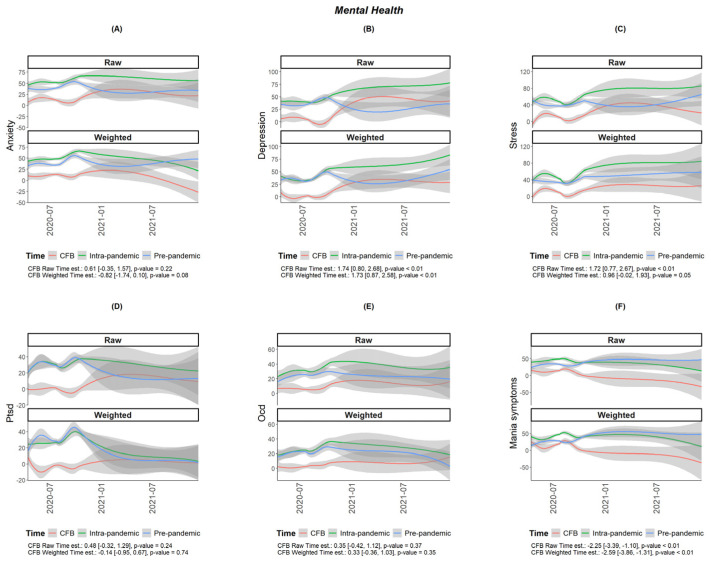
Plots of mental health outcomes of adult Cypriots (N = 917) on the Collaborative Outcome Study on Health and Functioning During Infection Times (COH-FIT) questionnaire [(**A**): Anxiety, (**B**): Depression, (**C**): Stress, (**D**): Post-traumatic symptoms, (**E**): Obsessive-compulsive symptoms, (**F**): Mania]. Symptoms presented as raw and weighted data, applying the raking method. Green lines show the intra-pandemic period, blue lines show pre-pandemic period, and red lines show the change from baseline as functions of time. The gray lines show the confidence intervals (95% CIs) and are darker when they overlap. *p*-values for the association between time and CFB are also displayed alongside the corresponding coefficients and 95% confidence intervals; coefficients’ estimates and confidence intervals denote changes per month.

**Figure 2:**
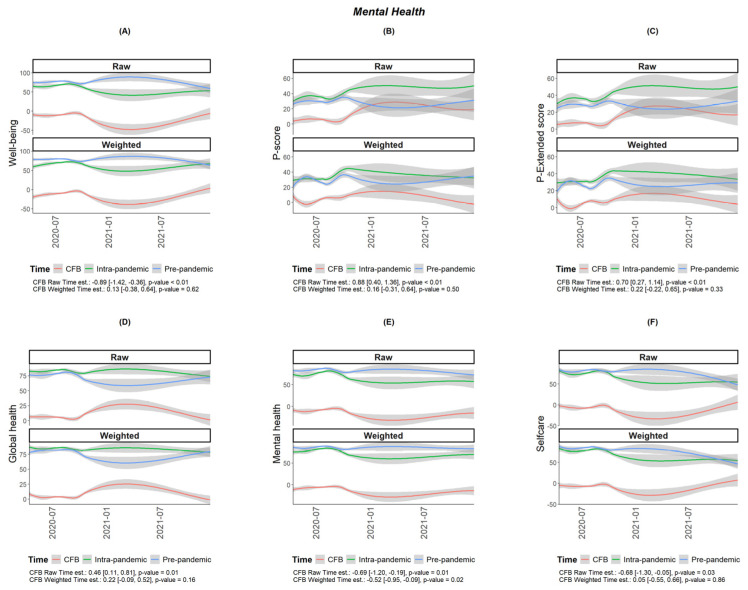
Plots of health outcomes of adult Cypriots (N = 917) on the Collaborative Outcome Study on Health and Functioning During Infection Times (COH-FIT) questionnaire [(**A**): Well-being, (**B**): P-score, (**C**): P-extended score, (**D**): Global health, (**E**): Mental health, (**F**): Self-care] presented as raw and weighted data, after applying the raking method. Green lines show the intra-pandemic period, blue lines show pre-pandemic period, and red lines show the change from baseline as functions of time. The gray lines show the confidence intervals (95% CIs) and are darker when they overlap. *p*-values for the association between time and CFB are also displayed alongside the corresponding coefficients and 95% confidence intervals; coefficients’ estimates and confidence intervals denote changes per month.

The COH-FIT scores related to global health and various aspects of mental health and behavior, including WHO-5-measured well-being and psychopathology, as measured with the p-score during versus pre-pandemic period, are shown in Table 1. The questionnaire responses show that both global health and mental health status, as depicted in Table 1, worsened significantly during the pandemic (5.59; 95% CI: 4.55 to 6.63 and −7.55; 95% CI −9.01 to −6.07, respectively). Analyzing the survey results, as shown in Table 1 and Figure 1 and Figure 2, mental health components worsened significantly, including anxiety (12.05; 95% CI: 9.33 to 14.77), stress (9.62; 95% CI: 7.08 to 12.15), and depression (4.60; 95% CI: 2.06 to 7.14), with a significant increase in the scores during the pandemic (*p* < 0.001). A significant rise was recorded in mood symptoms, with mania symptoms (14.85; 95% CI: 11.64 to 18.07) and mood swings (14.38; 95% CI: 11.92 to 16.84) being particularly affected (*p* < 0.001). Obsessive-compulsive symptoms also increased (3.14; 95% CI: 1.14 to 5.14), as did panic symptoms (0.22; 95% CI: 0.08 to 0.36), while suicidal attempts appeared to have decreased (−0.02; 95% CI: −0.05 to 0.00).

Taking into consideration the time of completion of the questionnaire, the scores on anxiety, depression, stress, and obsessive-compulsive symptoms, which were initially high, slightly increased over time, as reported for both the pre-pandemic and intra-pandemic COVID-19 period but were consistently higher during the COVID-19 study period, worsening with time, with the exception of mania symptoms (Figure 1). 

The changes in specific psychological and mood dimensions reported throughout the intra-pandemic period are shown in Table 1. Feelings such as anger, fear, helplessness, frustration, and stress increased (*p* < 0.001), as did feelings of boredom and loneliness (*p* < 0.001), while resilience significantly decreased *p* < 0.001). 

Mood swing and concentration difficulty were higher intra-pandemic than pre-pandemic (Figure S1). Suicide attempt scores were higher pre-pandemic than intra-pandemic, but self-injury and panic symptoms did not change significantly (Figure S1B–D). Both raw and weighted results were very similar for all items, but from the CFB, the time differences were significant for most of the outcomes according to the raw results but not the weighted results. 

Feelings like boredom, frustration, loneliness, and sleep problems showed higher scores intra-pandemic, increasing with time (Figure S2A–D). Resilience and aggressive acts had higher scores pre-pandemic than intra-pandemic (Figure S2G,H). 

### 3.3. Addictive Behaviors

The changes in addictive behaviors following the institution of restrictive measures instituted to minimize the impact of the COVID-19 pandemic are shown in Table 2. During the pandemic, there was a moderate increase in alcohol consumption (0.18; 95% CI: 0.01 to 0.35), substance use (0.06; 95% CI: 0.00 to 0.11), and gaming (0.30; 95% CI: 0.19 to 0.41). For gambling and consumption of tobacco and cannabis, no significant change was reported. Gambling, tobacco use, substance use, and gaming appeared to remain steady as time passed, as depicted in Figure S3. In contrast, cannabis use showed a statistically significant upswing towards the later months of the survey responses, according to CFB for both weighted and raw data (Figure S3C).

### 3.4. Physical Activity and Daily Activities

The scores of the participants on a series of daily activities during versus the pre-pandemic period in Cyprus are shown in Table 3. A significant increase in the use of social media, the Internet, and television (*p* < 0.001) was reported. 

A decrease in engagement in hobbies (−13.49; 95% CI: −16.05 to −10.94) and sports activities (−2.21; 95% CI: −4.37 to −0.05) was observed, as well as an overall decline in well-being and self-care (−11.06; 95% CI: −12.69 to −9.45 and −7.10; CI: −9.00 to −5.20). A significant negative impact on sleep and concentration difficulty was also observed.

Social media use and internet usage showed an increase during the pandemic but remained stable with time (Figure S4A,B). TV watching was higher during COVID-19 and showed an increase with time, while reading was lower during the pre-pandemic period, although increasing as time passed (Figure S4C,D). Musical activity was almost the same before and during the pandemic period (Figure S4E). The raw and weighted results were similar, but according to CFB indicated that the changes over time were not statistically significant.

The changes in daily activities during the COVID-19 measures had no apparent impact on the physical health of the study participants (Figure S5). The BMI was slightly lower in the pre-pandemic period and showed a decrease with time (Figure S5C). Sports activities were higher in the pre-pandemic period, and hobby activities and physical health were both higher in the pre-pandemic period than during COVID-19 and showed a decrease over time (Figure S5A,B,D). The raw and weighted results were very similar when graphically depicted, but according to CFB, the only item with a statistically significant change in both weighted and raw data was sports activity.

### 3.5. Consequences on Social Life

Table 4 shows the changes in social life and social support recorded during the pre- and intra-pandemic periods. There was a significant negative effect on family satisfaction (*p* < 0.001). An increase in family dysfunction (−7.97; 95% CI: −9.90 to −6.05) and an overall decline in social relations and social support (*p* < 0.001) were observed. Additionally, there was a statistically significant decrease in work functioning (−13.35; 95% CI: −15.52 to −11.18). The scores on family satisfaction, household satisfaction, social function, social support, and prosocial behavior were all higher in the pre-pandemic than the intra-pandemic period, as depicted graphically in Figure S6, but the differences were statistically significant only for family satisfaction and social function, for both raw and weighted results. 

## 4. Discussion

This online COH-FIT survey study demonstrates the significant toll that the COVID-19 pandemic, and the related COVID-19 pandemic measures introduced in Cyprus to contain it, has taken on the people of Cyprus, with a significant impact on their mental health and overall well-being. The participants, all adults, reported experiencing raised levels of anxiety, depression, and stress, accompanied by adverse changes in coping strategies and lifestyle choices. The study participants’ reliance on digital platforms, substance use, and alcohol consumption increased, while physical activity and daily activities associated with social support and self-care declined significantly. 

These findings are consistent with a growing body of documentation of the psychological repercussions of this global health crisis secondary to COVID-19 [25,26,27]. A national survey conducted in June 2020 in the United States found that 26.9% of parents and 14.3% of children experienced deterioration in their mental health during the COVID-19 pandemic [28] and that the impact on physical health was comparatively less, but also significant [28]. 

The COVID-19 Mental Disorders Collaborators documented a significant global rise in major depressive disorders (27.6%) and anxiety disorders (25.6%) in 2020, attributing these findings to the pandemic [29]. Decades of trauma research have revealed that after negative life events, such as loss or disasters, most individuals either exhibit resilience, with minimal impact on anxiety or depression symptoms or undergo a recovery process, showing an initial brief increase in symptoms followed by improvement [30].

The findings of the Cypriot COH-FIT study are in alignment with other studies, which indicate concerns regarding a decrease in the risk of suicide but not self-harm during the pandemic. There was no clinically meaningful change. A variety of factors, including social isolation, financial difficulties, and mental health challenges, collectively contribute to this increased risk [8,9,10], and according to one meta-analysis, the combined prevalence of suicidal ideation during the COVID-19 pandemic was 12.1% [30]. The rate of suicidal thoughts during COVID-19 surpassed pre-pandemic levels in the general population, signaling a potential increase in future suicide rates [31]. Several factors were associated with the presence of suicidal thoughts, including a low level of social support, high levels of physical and mental exhaustion, and poorer self-reported physical health in frontline medical workers, along with sleep disturbances, quarantine and exhaustion, loneliness, and mental health difficulties [31]. The findings on mental health status during the COVID-19 pandemic in Cyprus are in agreement with and reinforce the current literature [11,12,13], specifically the report of a rise in the prevalence of obsessive-compulsive symptoms. Individuals with OCD and related symptoms commonly experience obsessions centered around infection and hygiene. The fear of contracting or transmitting the virus can intensify these obsessions, increasing anxiety and triggering a surge in compulsive behaviors [32]. Regarding sleep, the Cypriot study indicated a significant increase in sleep problems, which is in agreement with other reports of poor quality of sleep and negative changes in sleep patterns [33,34,35]. Nevertheless, the literature is still scarce on this subject, with most available studies relying on subjective reports rather than objective criteria to assess sleep during the pandemic.

According to Robinson and colleagues [36], in the initial months of the COVID-19 pandemic (March–April 2020), a significant rise in mental health symptoms was observed, with a standardized mean change (SMC) of 0.102 (95% CI: 0.026 to 0.192). This increase gradually diminished over time, becoming non-significant in the period from May to July 2020 (SMC = 0.067, 95% CI: −0.022 to 0.157). When comparing different mental health indices, it was found that while anxiety (SMC = 0.13, *p* = 0.02) exhibited some change and general mental health did not change significantly (SMC = −0.03, *p* = 0.65), the increase in symptoms related to depression and mood disorders was more prominent, remaining significantly elevated in the May–July 2020 period (SMC = 0.20, 95% CI: 0.099 to 0.302). In primary analyses, the surge in mental health symptoms was particularly marked among individuals with pre-existing physical health conditions. Notably, at least in that study [36], no discernible shift in symptoms was reported among those with pre-existing mental health conditions. Overall, however, following the onset of the COVID-19 pandemic, there was a slight rise in mental health symptoms, which subsequently diminished and reached levels similar to those seen before the pandemic by the middle of 2020 [36].

The significant increase in the utilization of social media, the Internet, television, gaming, and substance use, including alcohol consumption, revealed in the Cypriot COH-FIT study, reflects shifts in coping mechanisms, lifestyle behaviors, and leisure activities in response to the pandemic and pandemic measures.

These changes could be attributed to the various challenges posed by the pandemic, such as prolonged periods of social isolation, economic uncertainty, and the disruption of daily routines. Individuals may have turned to these outlets as a way to navigate stress and seek solace [37,38]. A study conducted by Vanderbruggen and colleagues (2020) [39] on the impact of pandemic measures on alcohol consumption and smoking habits produced findings in agreement with the Cypriot COH-FIT results regarding increased use of alcohol, but not tobacco and smoking. These authors found a slight increase in alcohol consumption and a marginal rise in cigarette smoking compared with the period before the COVID-19 pandemic, and there are other reports of a 67% increase in alcohol sales during the pandemic and confinement [40,41]. Conversely, one survey showed a reduction in smoking and alcohol consumption during the pandemic [33]. The Cypriot COH-FIT study showed no significant difference in tobacco, cannabis, and gambling during the intra-pandemic period versus the pre-pandemic period, and the increased alcohol consumption may be related to factors such as increased stress, anxiety, boredom, and social isolation, which highlights the need for targeted interventions and support [42]. As Cypriots found themselves confined to their homes during the pandemic, there was a significant surge in the use of the Internet, social media, TV, and gaming. This increased reliance on the Internet raised concerns about problematic and addictive internet use and its impact on mental health. One narrative review aimed to provide an overview of the prevalence of problematic internet use during the pandemic, with a specific focus on three common types, namely online gaming, gambling, and pornography viewing [42]. The Cypriot results are in partial agreement with the current literature [18,43,44]. Sallis and colleagues (2021) [45] and Han and colleagues (2022) [33] published studies on the association between physical inactivity and severe COVID-19 outcomes. A study targeting an elderly population reported that physical inactivity was significantly associated with a higher risk of severe COVID-19 outcomes, independently of other risk factors. This emphasizes the importance of regular physical activity in reducing the risk of severe COVID-19 outcomes, and also as a therapeutic approach to combat the mental and physical consequences of COVID-19 and related pandemic measures, with a special emphasis on older individuals, and the potential benefits of exercise in maintaining physical and mental well-being [46]. The decrease in engagement in hobbies and sports observed in the Cypriot sample indicates a decline in physical activity and overall self-care. There is a widespread belief that engaging in physical activity and exercise exerts a favorable effect on mood and anxiety, and studies consistently link physical activity with an overall sense of well-being and improved mood, specifically addressing anxiety [47,48]. The findings of the Cypriot COH-FIT study underscore the interconnectedness of the various aspects of the lives of individuals and the multifaceted effects of the pandemic on their global health and well-being.

The pandemic caused major disruptions in daily lives, but there is still much to learn about the effects of social isolation during the COVID-19 pandemic, where social distancing was a key public health measure [47,48]. Observation of social isolation remains the safest way to prevent transmission of infection, but social isolation and loneliness have been linked to worsening of physical and mental health indices and higher mortality rates. The research findings of the Cypriot COH-FIT survey are in line with the international literature, as the social life of Cypriots appears to have been curtailed during the COVID-19 pandemic. 

The significant reduction in social interchange reported during the pandemic, including worsening in social support, social function, and family function, indicates a significant strain on social connections. The pandemic measures disrupted traditional social interactions and communal support systems, which are crucial for mental and emotional well-being. The findings of the Cypriot COH-FIT study highlight the relevant consequences of the pandemic on a variety of aspects of individual lives, including family dynamics, social connections, and the work environment.

## 5. Limitations

The Cypriot COH-FIT survey has several limitations. The cross-sectional design implies that data were collected from each participant at a single point in time during the pandemic, with a retrospective recall of the pre-pandemic status posing challenges in establishing causal relationships between variables. Longitudinal studies that track changes over time could provide deeper insights into the effects of the pandemic on mental and physical health. The anonymous design can provide more honest responses but limits the ability to verify the accuracy of self-reported data. Respondents may provide inaccurate information due to concerns about privacy or social desirability bias. A considerable percentage of participants chose not to respond to all the questions, and non-response bias could potentially affect the generalizability of the findings if non-respondents differ systematically from respondents in ways that are relevant to the study outcomes. While our goal was to recruit participants from all the various regions of Cyprus, it is possible that inherent biases within the sample collected via various outreach methods could affect the extent to which the results can be generalized to the broader population of Cypriots. The study relied on self-reported data, which may be subject to recall bias or misinterpretation. Objective measures and clinical assessments could provide more accurate data, especially when studying such topics as mental health. The study may not have accounted for all external factors that could influence the outcomes. Factors such as pre-existing mental health conditions or access to mental health services could confound the results. While the study identifies trends in behaviors (e.g., increased alcohol consumption), it could not delve deeply into the underlying reasons or motivations behind these behavioral changes. The study findings may not be fully generalizable to other populations or regions due to cultural, socioeconomic, or other contextual differences. The study data were collected during a specific time frame, and the situation regarding the pandemic and public health measures may have evolved since then, and the findings may not reflect the current state of affairs. Finally, we also acknowledge statistical limitations, such as an increased probability of finding a false positive result due to multiple tests and accepting an MCAR missing mechanism with high missing rates in some items.

## 6. Conclusions

In conclusion, according to the Cyprus COH-FIT study results, the COVID-19 pandemic has had a profound impact on the mental health of the Cypriot population. The challenges brought about by the pandemic and related paned measures, including fear of infection, social isolation, and economic uncertainty, contributed to an increase in anxiety, depression, and other adverse mental health conditions. It is crucial to prioritize mental health support and resources in order to address the long-lasting effects of the pandemic on individual well-being. There is an urgent need for targeted public health strategies that encompass both psychological and physical well-being and mental health support. Understanding the factors associated with the adverse changes that took place during the pandemic is vital for designing effective forms of intervention to restore and enhance the global health and resilience of the population. Only a few studies have currently reported original, nationally/geographically comparable, comprehensive, and multilingual representative data on health and well-being during the COVID-19 pandemic, and even fewer included person-level assessments from the pre-COVID-19 period, as the COH-FIT study aims to do.

In the future, epidemiological studies should prioritize investigating variations in psychopathology and the temporal aspects of mental health problems within diverse populations. Interventions need to be developed and implemented that address the prevailing psychosocial challenges and actively promote mental well-being during the COVID-19 pandemic.

Such interventions should address the pre-existing health issues and disparities that were exacerbated by the COVID-19 crisis. Immediate action is essential to prevent long-term consequences and to cultivate resilience in the face of this global health crisis.

## Figures and Tables

**Table 1:** Effects on mental health of adult Cypriots (N = 917) (weighted dependent samples *t*-test).

Outcome	Pre-Pandemic COVID-19 Measures Weighted Mean (sd)	Intra-Pandemic COVID-19 Measures Weighted Mean (sd)	Difference	95% CI	*p*-Value	Number of Missing (%)
Well-being	79.00 (15.52)	67.94 (22.31)	−11.06	(−12.69, −9.45)	<0.001	293 (31.95%)
P-score	28.52 (17.80)	34.07 (20.40)	5.55	(4.13, 6.97)	<0.001	357 (38.93%)
P-extended score	27.03 (17.43)	33.77 (19.62)	6.74	(5.45, 8.02)	<0.001	357 (38.93%)
Global health	79.21 (17.83)	84.80 (14.45)	5.59	(4.55, 6.63)	<0.001	230 (25.08%)
Mental health	86.54 (17.96)	78.99 (22.23)	−7.55	(−9.01, −6.07)	<0.001	243 (26.50%)
Selfcare	84.07 (19.22)	76.97 (25.06)	−7.10	(−9.00, −5.20)	<0.001	308 (33.59%)
Anxiety	39.82 (28.44)	51.87 (27.65)	12.05	(9.33, 14.77)	<0.001	375 (40.89%)
Depression	36.33 (28.23)	40.93 (29.43)	4.60	(2.06, 7.14)	<0.001	383 (41.77%)
Stress	37.96 (28.76)	47.58 (33.39)	9.62	(7.08, 12.15)	<0.001	431 (47.00%)
Post-traumatic symptoms	30.49 (29.63)	28.56 (26.26)	−1.93	(−4.27, 0.41)	0.106	386 (42.09%)
Obsessive-compulsive symptoms	23.49 (25.67)	26.63 (26.84)	3.14	(1.14, 5.14)	0.002	394 (42.97%)
Mania symptoms	29.94 (27.88)	44.79 (28.92)	14.85	(11.64, 18.07)	<0.001	411 (44.82%)
Mood swing	25.22 (24.65)	39.60 (31.99)	14.38	(11.92, 16.84)	<0.001	399 (43.51%)
Self-injury	0.02 (0.17)	0.02 (0.21)	0.00	(−0.02, 0.02)	0.761	410 (44.71%)
Panic symptoms	0.16 (0.78)	0.38 (1.57)	0.22	(0.08, 0.36)	0.002	411 (44.82%)
Suicide attempt	0.02 (0.23)	0.00 (0.00)	−0.02	(−0.05, −0.00)	0.018	416 (45.37%)
Anger	25.46 (25.13)	38.38 (32.41)	12.92	(10.54, 15.29)	<0.001	428 (46.67%)
Helpless	17.06 (21.7)	26.72 (31.74)	9.66	(7.25, 12.07)	<0.001	433 (47.22%)
Concentration difficulties	25.25 (26.48)	32.94 (31.93)	7.69	(5.58, 9.80)	<0.001	437 (47.66%)
Fear	18.05 (23.7)	40.30 (31.51)	22.25	(19.25, 25.26)	<0.001	431 (47.00%)
Bored	24.38(26.24)	43.61 (36.58)	19.23	(16.29, 22.18)	<0.001	434 (47.33%)
Frustration	27.49 (26.91)	40.81 (34.17)	13.32	(10.72, 15.93)	<0.001	434 (47.33%)
Loneliness	21.35 (27.25)	33.88 (35.09)	12.53	(9.94, 15.11)	<0.001	432 (47.11%)
Sleep problems	28.83 (27.99)	35.20 (31.37)	6.37	(4.08, 8.65)	<0.001	426 (46.46%)
Hallucination	9.70 (19.01)	9.13 (18.82)	−0.57	(−2.13, 0.98)	0.468	438 (47.76%)
Delusion	10.88 (20.47)	11.76 (22.29)	0.88	(−0.71, 2.46)	0.279	437 (47.66%)
Resilience	79.22 (19.66)	71.25 (22.77)	−7.97	(−9.56, −6.38)	<0.001	216 (23.56%)
Aggression act	0.07 (0.72)	0.06 (0.47)	−0.01	(−0.06, 0.05)	0.839	413 (45.04%)

CI = confidence interval. Significance level *p* < 0.05.

**Table 2:** Collaborative Outcome Study on Health and Functioning During Infection Times (COH-FIT) in Cyprus: changes in addictive behaviors of adult Cypriots (N = 917) following COVID-19 measures, according to the COH-FIT questionnaire (weighted dependent samples *t*-test).

Outcome	Pre-Pandemic COVID-19 Measures Weighted Mean (sd)	Intra-Pandemic COVID-19 Measures Weighted Mean (sd)	Difference	95% CI	*p*-Value	Number of Missing (%)
Tobacco	3.14 (7.59)	3.28 (8.00)	0.14	(−0.14, 0.44)	0.324	443 (48.31%)
Alcohol	1.93 (2.88)	2.11 (3.29)	0.18	(0.01, 0.35)	0.038	441 (48.09%)
Cannabis	0.02 (0.23)	0.02 (0.24)	0.00	(−0.04, 0.02)	0.579	449 (48.96%)
Gamble	5.80 (16.89)	5.80 (16.98)	0.00	(−1.50, 1.50)	1.00	444 (48.42%)
Substances	0.10 (0.63)	0.16 (1.05)	0.06	(0.00, 0.11)	0.035	446 (48.64%)
Gaming	0.46 (0.88)	0.76 (1.61)	0.30	(0.19, 0.41)	<0.001	439 (47.87%)

CI = confidence interval. Significance level *p* < 0.05.

**Table 3:** Collaborative Outcome Study on Health and Functioning During Infection Times (COH-FIT) in Cyprus: Effects on physical and daily activity of adult Cypriots (N = 917), according to the COH-FIT questionnaire following COVID-19 measures (weighted dependent samples *t*-test).

Outcome	Pre-Pandemic COVID-19 Measures Weighted Mean (sd)	Intra-Pandemic COVID-19 Measures Weighted Mean (sd)	Difference	95% CI	*p*-Value	Number of Missing (%)
Social media	2.29 (2.79)	3.18 (3.24)	0.89	(0.71, 1.09)	<0.001	437 (47.66%)
Internet	2.37 (2.35)	3.23 (3.33)	0.86	(0.67, 1.04)	<0.001	444 (48.42%)
TV	1.90 (1.79)	2.76 (2.48)	0.86	(0.68, 1.04)	<0.001	444 (48.42%)
Reading	1.81 (2.49)	1.98 (2.59)	0.17	(−0.01, 0.35)	0.063	448 (48.85%)
Music	2.05 (3.32)	1.91 (2.45)	−0.14	(−0.40, 0.10)	0.235	444 (48.42%)
Sports	24.03 (34.06)	21.82 (28.67)	−2.21	(−4.37, −0.05)	0.045	437 (47.66%)
BMI	25.34 (4.34)	25.50 (4.49)	0.16	(0.07, 0.24)	0.001	215 (23.45%)
Hobbies	77.79 (24.30)	64.30 (31.16)	−13.49	(−16.05, −10.94)	<0.001	314 (34.24%)
Physical health	83.26 (16.13)	79.81 (19.18)	−3.45	(−4.59, −2.32)	<0.001	247 (26.94%)

**Table 4:** Collaborative Outcome Study on Health and Functioning During Infection Times (COH-FIT) Cyprus study: Changes in the social life of adult Cypriots (N = 917) following COVID-19 measures, according to the COH-FIT questionnaire (weighted dependent samples *t*-test).

Outcome	Pre-Pandemic COVID-19 Measures Weighted Mean (sd)	Intra-Pandemic COVID-19 Measures Weighted Mean (sd)	Difference	95% CI	*p*-Value	Number of Missing (%)
Family satisfaction	81.86 (20.71)	73.89 (25.88)	−7.97	(−9.90, −6.05)	<0.001	193 (21.05%)
Household satisfaction	82.54 (21.27)	82.63 (21.56)	0.09	(−1.73, 1.92)	0.921	280 (30.53%)
Social function	83.06 (17.81)	71.79 (25.69)	−11.27	(−13.19, −9.35)	<0.001	313 (34.13%)
Work functioning	82.75 (19.86)	69.40 (27.97)	−13.35	(−15.52, −11.18)	<0.001	314 (34.24%)
Social support	4.62 (7.24)	3.90 (5.62)	−0.72	(−1.09, −0.34)	<0.001	425 (46.35%)
Prosocial behavior	2.25 (2.85)	2.24 (3.23)	−0.01	(−0.22, 0.19)	0.874	432 (47.11%)

## Data Availability

The raw data supporting the conclusions of this article will be made available by the authors on request.

## Supplementary Materials

The following supporting information can be downloaded at: https://www.mdpi.com/article/10.3390/jcm13185395/s1.

## References

[1] Dong E., Du H., Gardner L. (2020). An Interactive Web-Based Dashboard to Track COVID-19 in Real Time. Lancet Infect. Dis..

[2] Coronavirus Disease (COVID-19)—World Health Organization. Who.Int. https://www.who.int/emergencies/diseases/novel-coronavirus-2019?adgroupsurvey={adgroupsurvey}&gclid=cj0kcqjwunembhcbarisadp74qr2zd-flmmadmdl40fpfylqtwmy4nv9xbbo81p--3r1q4bixjlfimkaalyqealw_wcb.

[3] ArcGIS Dashboards Arcgis.Com. https://www.arcgis.com/apps/dashboards/bda7594740fd40299423467b48e9ecf6.

[4] Solmi M., Estradé A., Thompson T., Agorastos A., Radua J., Cortese S., Dragioti E., Leisch F., Vancampfort D., Thygesen L.C. (2022). Physical and Mental Health Impact of COVID-19 on Children, Adolescents, and Their Families: The Collaborative Outcomes Study on Health and Functioning during Infection Times—Children and Adolescents (COH-FIT-C&A). J. Affect. Disord..

[5] Wang C., Pan R., Wan X., Tan Y., Xu L., Ho C.S., Ho R.C. (2020). Immediate Psychological Responses and Associated Factors during the Initial Stage of the 2019 Coronavirus Disease (COVID-19) Epidemic among the General Population in China. Int. J. Environ. Res. Public Health.

[6] Alonso J., Vilagut G., Mortier P., Ferrer M., Alayo I., Aragón-Peña A., Aragonès E., Campos M., Cura-González I.D., Emparanza J.I. (2021). Mental Health Impact of the First Wave of COVID-19 Pandemic on Spanish Healthcare Workers: A Large Cross-Sectional Survey. Rev. Psiquiatr. Salud Ment..

[7] Shah S.M.A., Mohammad D., Qureshi M.F.H., Abbas M.Z., Aleem S. (2021). Prevalence, Psychological Responses and Associated Correlates of Depression, Anxiety and Stress in a Global Population, During the Coronavirus Disease (COVID-19) Pandemic. Community Ment. Health J..

[8] Motahedi S., Aghdam N.F., Khajeh M., Baha R., Aliyari R., Bagheri H., mardani A. (2021). Anxiety and Depression among Healthcare Workers during COVID-19 Pandemic: A Cross-Sectional Study. Heliyon.

[9] Wang S., Quan L., Chavarro J.E., Slopen N., Kubzansky L.D., Koenen K.C., Kang J.H., Weisskopf M.G., Branch-Elliman W., Roberts A.L. (2022). Associations of Depression, Anxiety, Worry, Perceived Stress, and Loneliness Prior to Infection With Risk of Post-COVID-19 Conditions. JAMA Psychiatry.

[10] Bakaloudi D.R., Barazzoni R., Bischoff S.C., Breda J., Wickramasinghe K., Chourdakis M. (2022). Impact of the First COVID-19 Lockdown on Body Weight: A Combined Systematic Review and a Meta-Analysis. Clin. Nutr..

[11] Ruissen M.M., Regeer H., Landstra C.P., Schroijen M., Jazet I., Nijhoff M.F., Pijl H., Ballieux B.E.P.B., Dekkers O., Huisman S.D. (2021). Increased Stress, Weight Gain and Less Exercise in Relation to Glycemic Control in People with Type 1 and Type 2 Diabetes during the COVID-19 Pandemic. BMJ Open Diabetes Res. Care.

[12] Wunsch K., Kienberger K., Niessner C. (2022). Changes in Physical Activity Patterns Due to the COVID-19 Pandemic: A Systematic Review and Meta-Analysis. Int. J. Environ. Res. Public Health.

[13] Dubey M.J., Ghosh R., Chatterjee S., Biswas P., Chatterjee S., Dubey S. (2020). COVID-19 and Addiction. Diabetes Metab. Syndr. Clin. Res. Rev..

[14] Chick J. (2020). Editorial: Alcohol and COVID-19. Alcohol Alcohol..

[15] Murthy P., Narasimha V.L. (2021). Effects of the COVID-19 Pandemic and Lockdown on Alcohol Use Disorders and Complications. Curr. Opin. Psychiatry.

[16] Roberts A., Rogers J., Mason R., Siriwardena A.N., Hogue T., Whitley G.A., Law G.R. (2021). Alcohol and Other Substance Use during the COVID-19 Pandemic: A Systematic Review. Drug Alcohol. Depend..

[17] Li Y.Y., Sun Y., Meng S.Q., Bao Y.P., Cheng J.L., Chang X.W., Ran M.S., Sun Y.K., Kosten T., Strang J. (2021). Internet Addiction Increases in the General Population During COVID-19: Evidence from China. Am. J. Addict..

[18] Gjoneska B., Potenza M.N., Jones J., Corazza O., Hall N., Sales C.M.D., Grünblatt E., Martinotti G., Burkauskas J., Werling A.M. (2022). Problematic Use of the Internet during the COVID-19 Pandemic: Good Practices and Mental Health Recommendations. Compr. Psychiatry.

[19] Solmi M., Estradé A., Thompson T., Agorastos A., Radua J., Cortese S., Dragioti E., Leisch F., Vancampfort D., Thygesen L.C. (2022). The Collaborative Outcomes Study on Health and Functioning during Infection Times in Adults (COH-FIT-Adults): Design and Methods of an International Online Survey Targeting Physical and Mental Health Effects of the COVID-19 Pandemic. J. Affect. Disord..

[20] Quattrocchi A., Mamais I., Tsioutis C., Christaki E., Constantinou C., Koliou M., Pana Z.D., Silvestros V., Theophanous F., Haralambous C. (2020). Extensive Testing and Public Health Interventions for the Control of COVID-19 in the Republic of Cyprus between March and May 2020. J. Clin. Med..

[21] ΠAΓΚOΣΜΙA ΜΕΛΕΤH ΥΓΕΙAΣ ΚAΙ ΛΕΙΤOΥΡΓΙΚOΤHΤAΣ ΣΕ ΠΕΡΙOΔOΥΣ ΜΕΤAΔOΤΙΚΩΝ ΛOΙΜΩΞΕΩΝ. Coh-fit.com. https://www.coh-fit.com/?lang=el.

[22] Solmi M., Thompson T., Estradé A., Agorastos A., Radua J., Cortese S., Dragioti E., Leisch F., Vancampfort D., Thygesen L.C. (2023). Validation of the Collaborative Outcomes Study on Health and Functioning during Infection Times (COH-FIT) Questionnaire for Adults. J. Affect. Disord..

[23] Deville J.C., Särndal C.E., Sautory O. (1993). Generalized Raking Procedures in Survey Sampling. J. Am. Stat. Assoc..

[24] Pasek J. ANES Raking Implementation; 2022. http://www.electionstudies.org.

[25] Deng J., Zhou F., Hou W., Silver Z., Wong C.Y., Chang O., Huang E., Zuo Q.K. (2021). The Prevalence of Depression, Anxiety, and Sleep Disturbances in COVID-19 Patients: A Meta-Analysis. Ann. N. Y Acad. Sci..

[26] Lee H., Choi D., Lee J.J. (2022). Depression, Anxiety, and Stress in Korean General Population during the COVID-19 Pandemic. Epidemiol. Health.

[27] Daly M., Robinson E. (2022). Depression and Anxiety during COVID-19. Lancet.

[28] Patrick S.W., Henkhaus L.E., Zickafoose J.S., Lovell K., Halvorson A., Loch S., Letterie M., Davis M.M. Well-Being of Parents and Children During the COVID-19 Pandemic: A National Survey. http://publications.aap.org/pediatrics/article-pdf/146/4/e2020016824/1080616/peds_2020016824.pdf.

[29] Santomauro D.F., Mantilla Herrera A.M., Shadid J., Zheng P., Ashbaugh C., Pigott D.M., Abbafati C., Adolph C., Amlag J.O., Aravkin A.Y. (2021). Global Prevalence and Burden of Depressive and Anxiety Disorders in 204 Countries and Territories in 2020 Due to the COVID-19 Pandemic. Lancet.

[30] Chen S., Bonanno G.A. (2020). Psychological Adjustment during the Global Outbreak of COVID-19: A Resilience Perspective. Psychol Trauma.

[31] Farooq S., Tunmore J., Ali W., Ayub M. (2021). Suicide, Self-Harm and Suicidal Ideation during COVID-19: A Systematic Review. Psychiatry Res..

[32] Prati G., Mancini A.D. (2021). The Psychological Impact of COVID-19 Pandemic Lockdowns: A Review and Meta-Analysis of Longitudinal Studies and Natural Experiments. Psychol. Med..

[33] Han Y.S., Yang H.Y., Ko Y. (2022). COVID-19-Related Anxiety and Lifestyle Changes. Front Public Health.

[34] Eleftheriou A., Rokou A., Arvaniti A., Nena E., Steiropoulos P. (2021). Sleep Quality and Mental Health of Medical Students in Greece During the COVID-19 Pandemic. Front Public Health.

[35] Datta K., Tripathi M. (2021). Sleep and COVID-19. Neurol. India.

[36] Robinson E., Sutin A.R., Daly M., Jones A. (2022). A Systematic Review and Meta-Analysis of Longitudinal Cohort Studies Comparing Mental Health before versus during the COVID-19 Pandemic in 2020. J. Affect. Disord..

[37] Zarreen Simnani F., Singh D., Choudhury A., Akhtar A. (2023). Impact of COVID-19 on Brain and Psychological Health, Its Possible Mechanisms, and Coping Strategies. Recent Pat. Biotechnol..

[38] Haider S.I., Ahmed F., Pasha H., Pasha H., Farheen N., Zahid M.T. (2022). Life Satisfaction, Resilience and Coping Mechanisms among Medical Students during COVID-19. PLoS ONE.

[39] Vanderbruggen N., Matthys F., Van Laere S., Zeeuws D., Santermans L., Van Den Ameele S., Crunelle C.L. (2020). Self-Reported Alcohol, Tobacco, and Cannabis Use during COVID-19 Lockdown Measures: Results from a Web-Based Survey. Eur. Addict. Res..

[40] Finlay I., Gilmore I. (2020). COVID-19 and Alcohol-a Dangerous Cocktail. BMJ.

[41] Sharma A., Kroumpouzos G., Lotti T., Goldust M. (2021). COVID-19 and Alcohol Use. Drug Alcohol Rev..

[42] Clay J.M., Parker M.O. (2020). Alcohol Use and Misuse during the COVID-19 Pandemic: A Potential Public Health Crisis?. Lancet Public Health.

[43] Pallavicini F., Pepe A., Mantovani F. (2022). The Effects of Playing Video Games on Stress, Anxiety, Depression, Loneliness, and Gaming Disorder during the Early Stages of the COVID-19 Pandemic: PRISMA Systematic Review. Cyberpsychol. Behav. Soc. Netw..

[44] Venegas A.V., Colbert G.B., Lerma E. (2020). Positive and Negative Impact of Social Media in the COVID-19 Era. Rev. Cardiovasc. Med..

[45] Sallis R., Young D.R., Tartof S.Y., Sallis J.F., Sall J., Li Q., Smith G.N., Cohen D.A. (2021). Physical Inactivity Is Associated with a Higher Risk for Severe COVID-19 Outcomes: A Study in 48 440 Adult Patients. Br. J. Sports Med..

[46] Jiménez-Pavón D., Carbonell-Baeza A., Lavie C.J. (2020). Physical Exercise as Therapy to Fight against the Mental and Physical Consequences of COVID-19 Quarantine: Special Focus in Older People. Prog. Cardiovasc. Dis..

[47] Grolli R.E., Mingoti M.E.D., Bertollo A.G., Luzardo A.R., Quevedo J., Réus G.Z., Ignácio Z.M. (2021). Impact of COVID-19 in the Mental Health in Elderly: Psychological and Biological Updates. Mol. Neurobiol..

[48] Heinberg L.J., Steffen K. (2021). Social Isolation and Loneliness During the COVID-19 Pandemic: Impact on Weight. Curr. Obes. Rep..

