# Supplementary material for: The Collaborative Outcome Study on Health and Functioning during Infection Times (COH-FIT): Results from Cyprus
Source: J Clin Med. 2024 Sep 12;13(18):5395. doi: 10.3390/jcm13185395 (PMC11432565; doi:10.3390/jcm13185395)
Supplement: Supplementary file 1 [file jcm-13-05395-s001.zip › jcm-3132189-supplementary.pdf]

## Supplementary Materials

**Table S1.** Demographic characteristics of the Collaborative Outcome Study on Health and Functioning During Infection Times (COH-FIT) study population in Cyprus (917 Adult Cypriots).

| Demographic characteristics | Sample | Weighted sample |
|---|---|---|
| **Age** | | |
| Mean (±SD) | 39.18 (±13.22) | 44.06 (±16.79) |
| Quartiles (Q1, Q3) | (29.00, 48.00) | (31.00, 58.00) |
| Missing (%) | 140 (15.3%) | |
| **Gender** | | |
| Male | 239 (31.0%) | 49.0% |
| Female | 527 (68.4%) | 51.0% |
| Non-binary | 4 (0.5%) | 0.0% |
| Transgender or intersex | 0 (0.0%) | 0.0% |
| Missing (%) | 147 (16.0%) | |
| **Ethnicity** | | |
| White | 736 (95.5%) | 95.2% |
| African/African-descent | 1 (0.1%) | 0.1% |
| Hispanic | 4 (0.5%) | 0.8% |
| Asian | 2 (0.3%) | 0.1% |
| Mixed | 13 (1.7%) | 1.7% |
| Other | 10 (1.3%) | 1.1% |
| Prefer not to answer | 5 (0.6%) | 1.0% |
| Missing (%) | 146 (15.9%) | |
| **Marital status** | | |
| Single never married | 275 (35.7%) | 33.8% |
| Married/ co-living partner | 443 (57.5%) | 60.4% |
| Widowed | 9 (1.2%) | 1.1% |
| Divorced or separated | 43 (5.6%) | 4.7% |
| Missing (%) | 147 (16.0%) | |
| **Educational level** | | |
| None | 1 (0.1%) | 0.0% |
| Primary school | 4 (0.5%) | 0.5% |
| High school | 157 (20.4%) | 21.9% |
| College/university degree | 552 (71.7%) | 68.0% |
| PhD | 56 (7.3%) | 9.6% |
| Missing (%) | 147 (16.0%) | |

**Table S2.** Collaborative Outcome Study on Health and Functioning During Infection Times (COH-FIT) in Cyprus: Employment status of the study population (917 Adult Cypriots).

| Employment status | Sample | Weighted sample |
|---|---|---|
| **Job** | | |
| No | 225 (29.5%) | 33.4% |
| Yes | 538 (70.5%) | 66.6% |
| Missing (%) | 154 (16.8%) | |
| **Lost job due to COVID-19** | | |
| No | 172 (77.1%) | 87.7% |
| Yes | 51 (22.9%) | 12.3% |
| Missing (%) | 2 (1.0%) | |
| **Job modality (smart working…)** | | |
| Going to work full-time | 347 (64.7%) | 65.8% |
| Going to work reduced time | 69 (12.9%) | 14.8% |
| Remote/home office | 90 (16.8%) | 16.0% |
| Forced vacation | 30 (5.6%) | 3.4% |
| Missing (%) | 2 (0.4%) | |

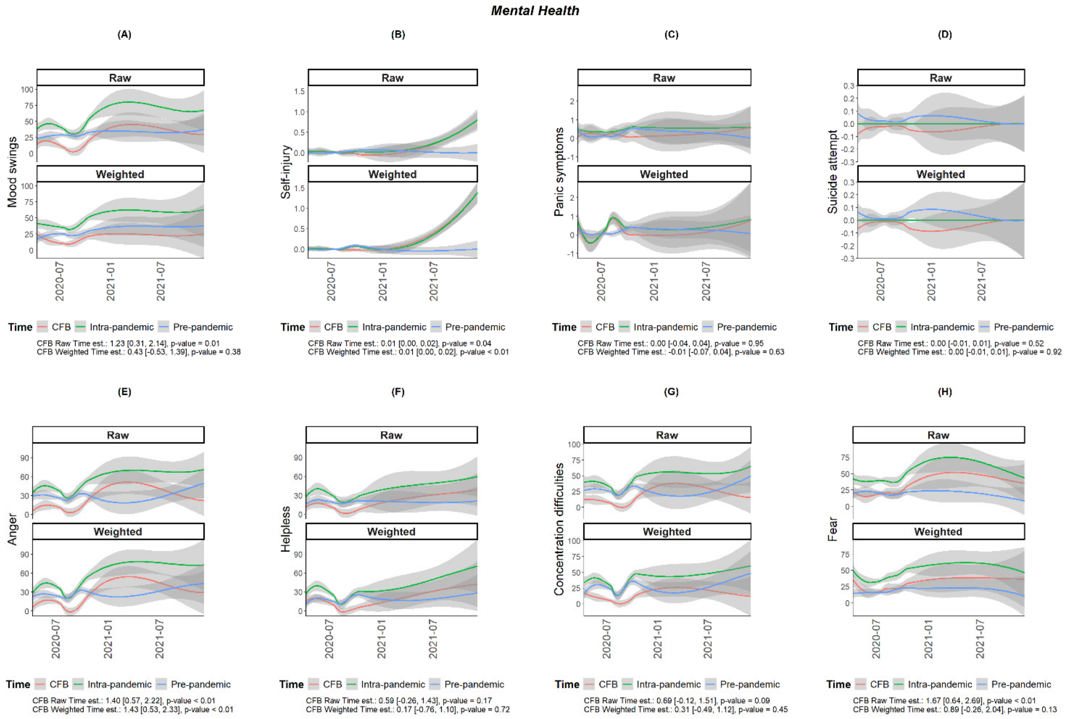


**Figure S1:** Plots of Mental Health outcomes of adult Cypriots (N=917) on the Collaborative Outcome Study on Health and Functioning During Infection Times (COH-FIT) questionnaire (Mood swings, Self-injury, Panic symptoms, Suicide attempt, Anger, Helpless, Concentration difficulties, and Fear), presented as Raw and Weighted data, applying the raking method. Green lines show the intra-pandemic period, blue lines show pre-pandemic period and red lines the change from baseline as functions of time. The gray lines show the confidence intervals (95% CIs) and are darker when they overlap. P-values for the association between time and CFB are also displayed, alongside with the corresponding coefficients and 95% confidence intervals; coefficients' estimates, and confidence intervals denotes changes per month.

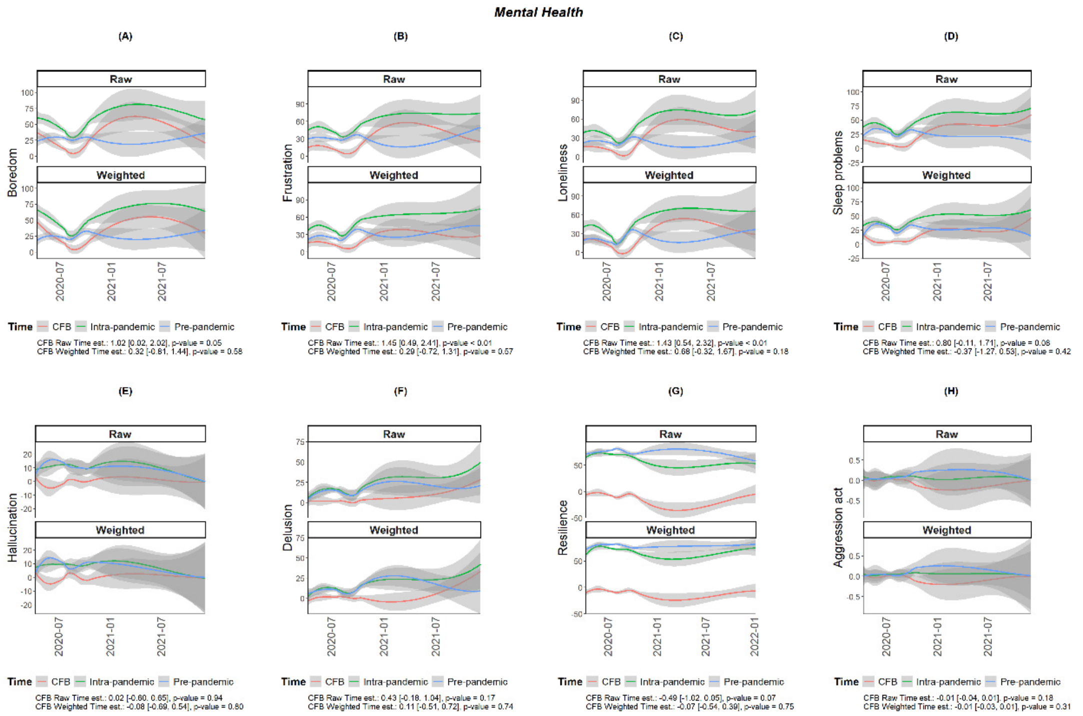


**Figure S2:** Plots of Mental Health outcomes of adult Cypriots (N=917) on the Collaborative Outcome Study on Health and Functioning During Infection Times (COH-FIT) questionnaire (Boredom, Frustration, Loneliness, Sleep problems, Hallucination, Delusion, Resilience, and Aggression act), presented as Raw and Weighted data, applying the raking method. Green lines show the intra-pandemic period, blue lines show pre-pandemic period and red lines the change from baseline as functions of time. The gray lines show the confidence intervals (95% CIs) and are darker when they overlap. P-values for the association between time and CFB are also displayed, alongside with the corresponding coefficients and 95% confidence intervals; coefficients' estimates, and confidence intervals denotes changes per month.

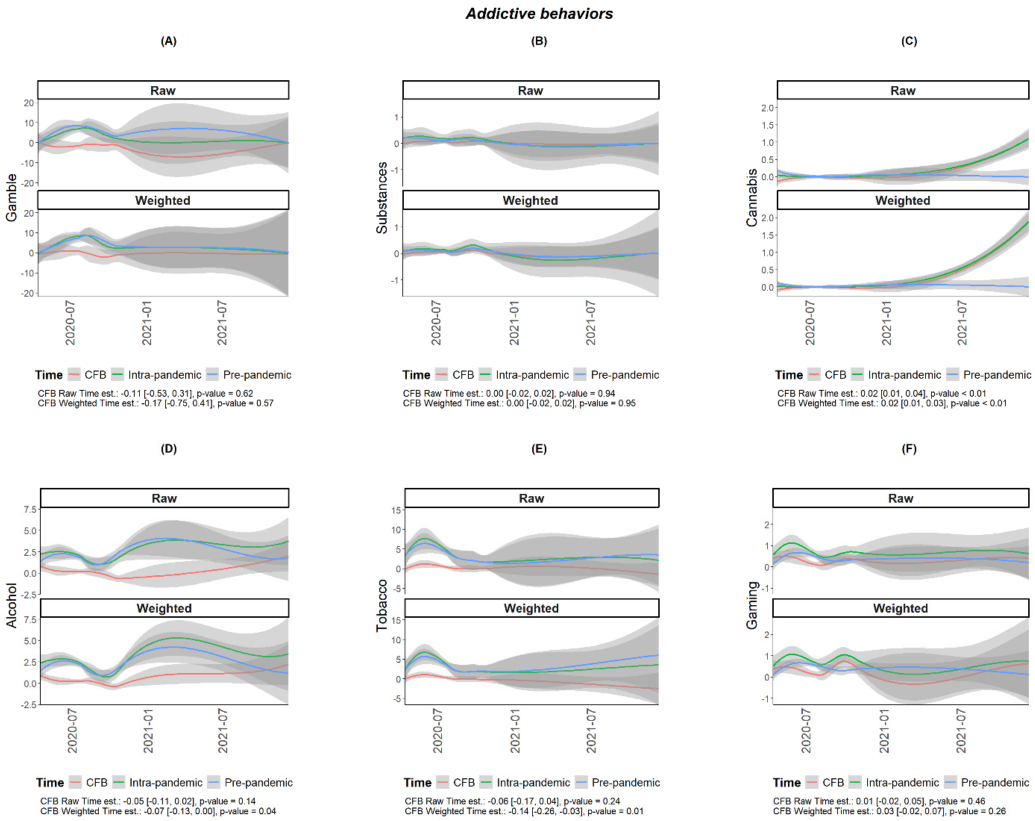


**Figure S3:** Plots of Addictive behaviors of adult Cypriots (N=917) on the Collaborative Outcome Study on Health and Functioning During Infection Times (COH-FIT) questionnaire (Gamble, Substances, Cannabis, Alcohol, Tobacco, Gaming) presented as Raw and Weighted data, applying the raking method. Green lines show the intra-pandemic period, blue lines show pre-pandemic period and red lines the change from baseline as functions of time. The gray lines show the confidence intervals (95% CIs) and are darker when they overlap. P-values for the association between time and CFB are also displayed, alongside with the corresponding coefficients and 95% confidence intervals; coefficients' estimates, and confidence intervals denotes changes per month.

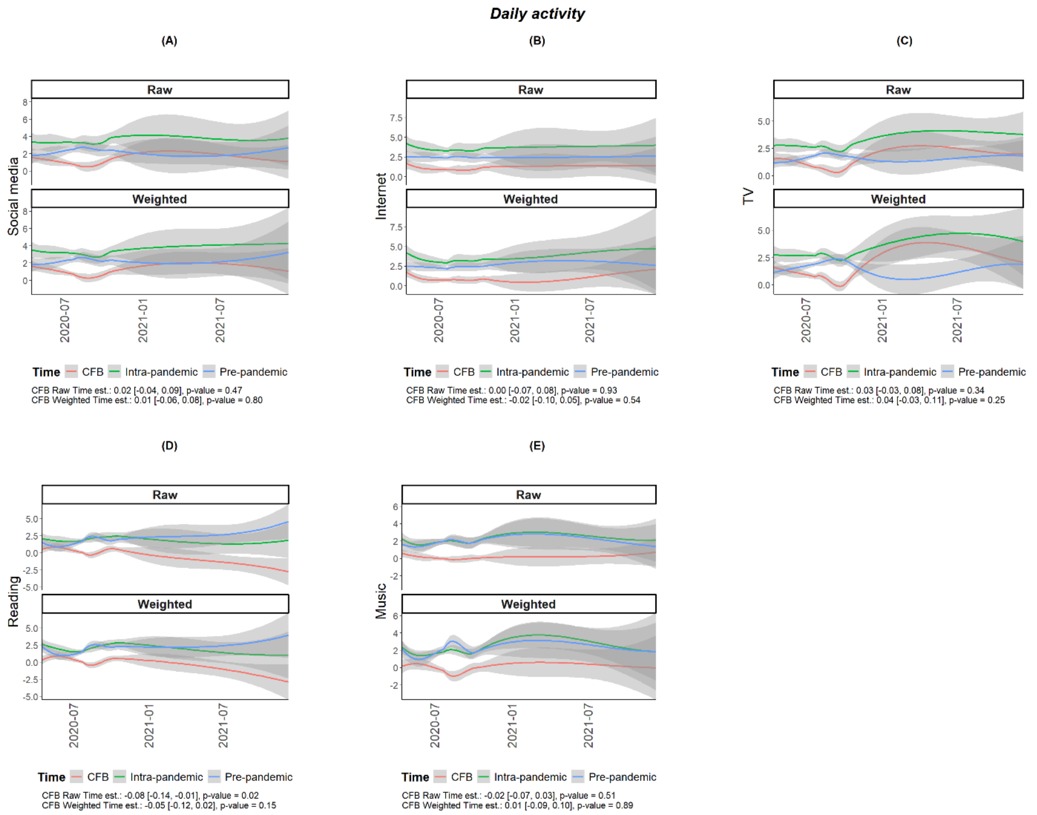


**Figure S4:** Plots of the Daily Activities of adult Cypriots (N=917) on the Collaborative Outcome Study on Health and Functioning During Infection Times (COH-FIT) questionnaire (Social media, Internet, TV, Reading, Music) presented as Raw and Weighted data, applying the raking method. Green lines show the intra-pandemic period, blue lines show pre-pandemic period and red lines the change from baseline as functions of time. The gray lines show the confidence intervals (95% CIs) and are darker when they overlap. P-values for the association between time and CFB are also displayed, alongside with the corresponding coefficients and 95% confidence intervals; coefficients' estimates, and confidence intervals denotes changes per month.

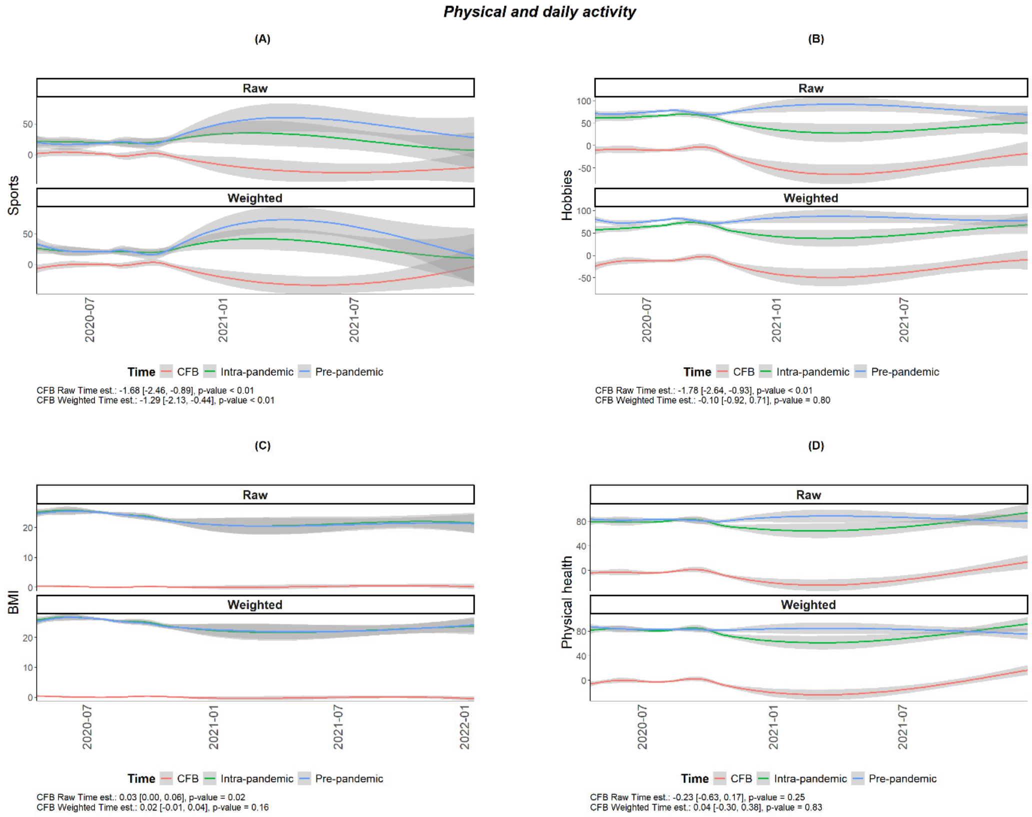


**Figure S5:** Plots of the Physical and daily activities of adult Cypriots (N=917) on the Collaborative Outcome Study on Health and Functioning During Infection Times (COH-FIT) questionnaire [Sports, Body Mass Index (BMI), Hobbies, Physical Health], presented as Raw and Weighted data, applying the raking method. Green lines show the intra-pandemic period, blue lines show pre-pandemic period and red lines the change from baseline as functions of time. The gray lines show the confidence intervals (95% CIs) and are darker when they overlap. P-values for the association between time and CFB are also displayed, alongside with the corresponding coefficients and 95% confidence intervals; coefficients' estimates, and confidence intervals denotes changes per month.

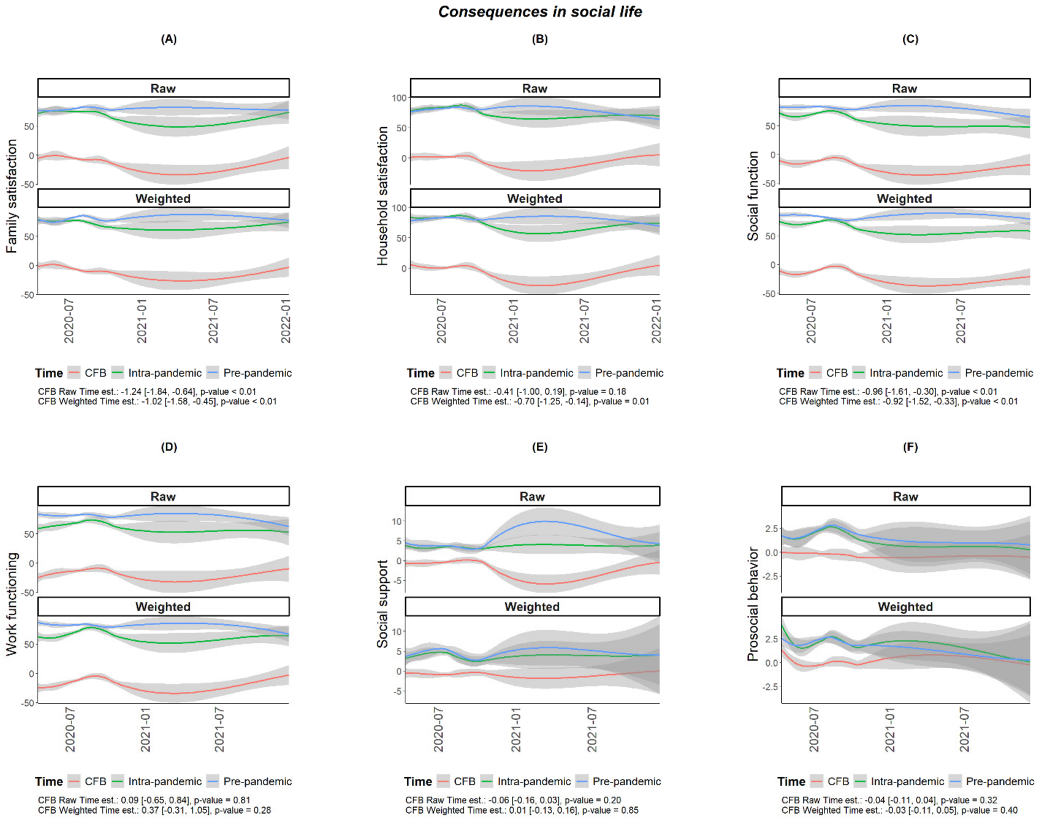


**Figure S6:** Plots of the Consequences on Social Life of adult Cypriots (N=917) on the Collaborative Outcome Study on Health and Functioning During Infection Times (COH-FIT) questionnaire (Family satisfaction, Household satisfaction, Social function, Work functioning, Social support, Prosocial behavior), presented as Raw and Weighted data, applying the raking method. Green lines show the intra-pandemic period, blue lines show pre-pandemic period and red lines the change from baseline as functions of time. The gray lines show the confidence intervals (95% CIs) and are darker when they overlap. P-values for the association between time and CFB are also displayed, alongside with the corresponding coefficients and 95% confidence intervals; coefficients' estimates, and confidence intervals denotes changes per month.

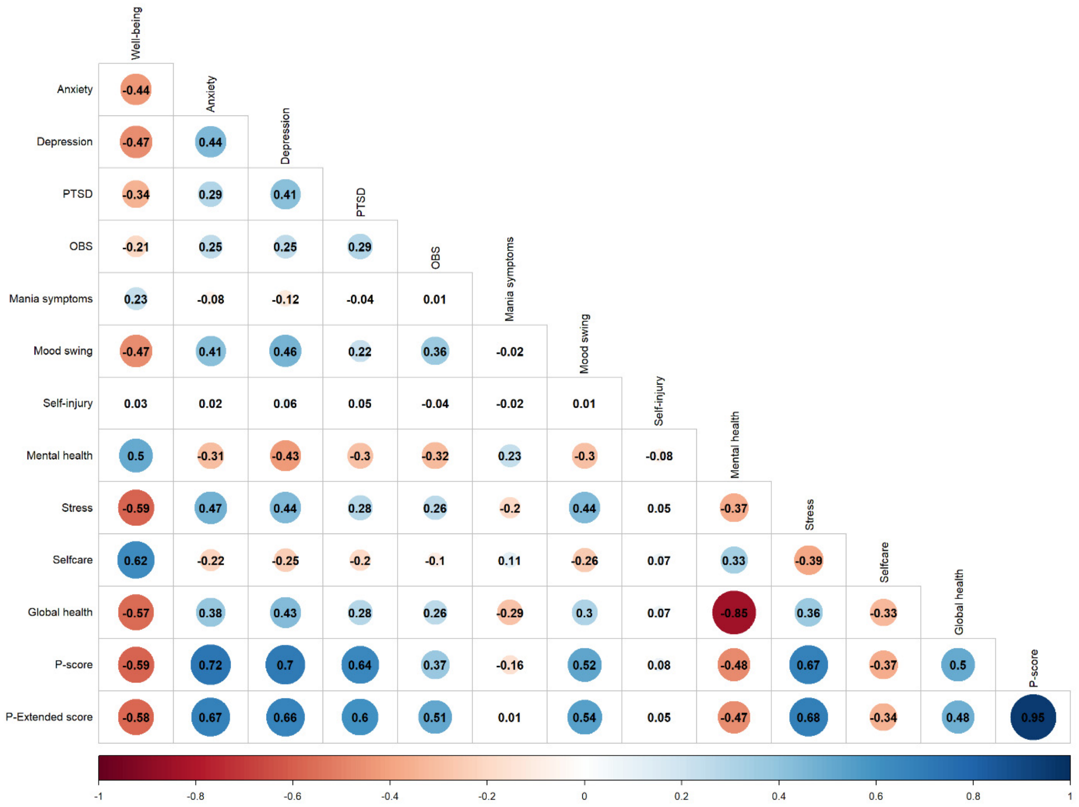


**Figure S7:** Weighted Pearson correlations table of the Well-being, P-score, P-Extended score, Global health, Selfcare, Stress, Mental health, Self-injury, Mood swing, Mania symptoms, OBS, PTSD, Depression, and Anxiety. The size and the color scale of the circle in the correlation table, express the magnitude, and the sign of the correlation, respectively.

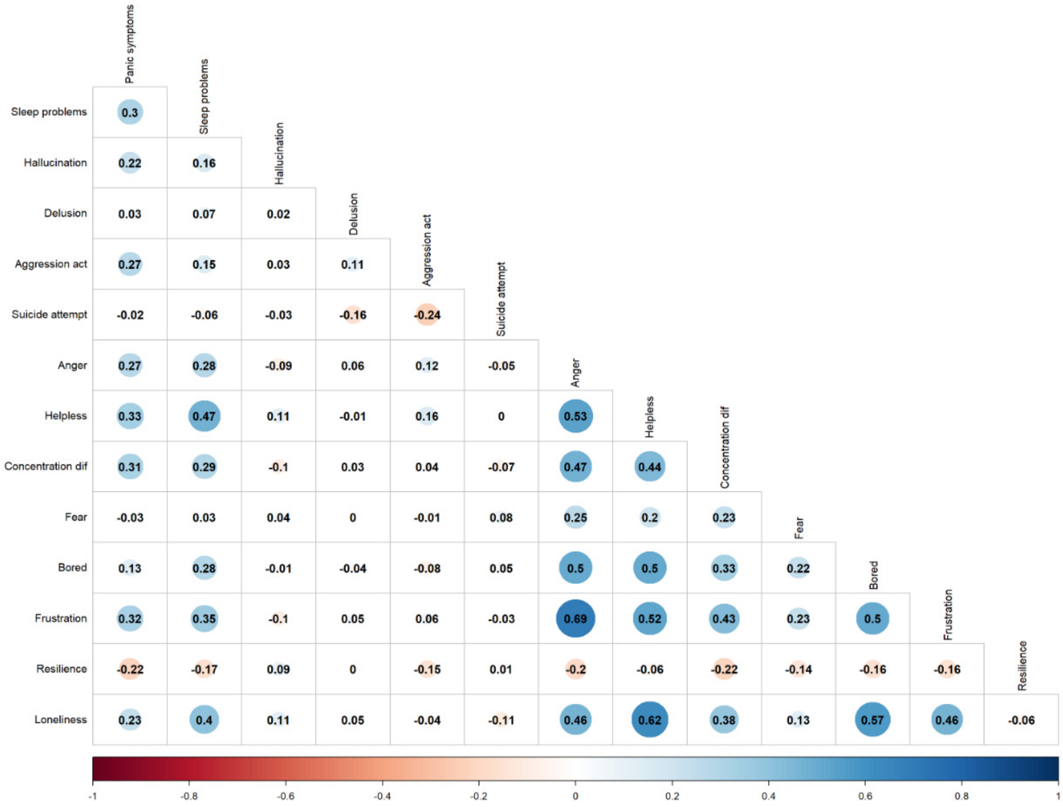


**Figure S8:** Weighted Pearson correlations table of the Loneliness, Resilience, Frustration, Bored, Fear, Concentration difficulties, Helpless, Anger, Suicide attempt, Aggression act, Delusion, Hallucination, Sleep problems, and Panic symptoms. The size and the color scale of the circle in the correlation table, express the magnitude, and the sign of the correlation, respectively.

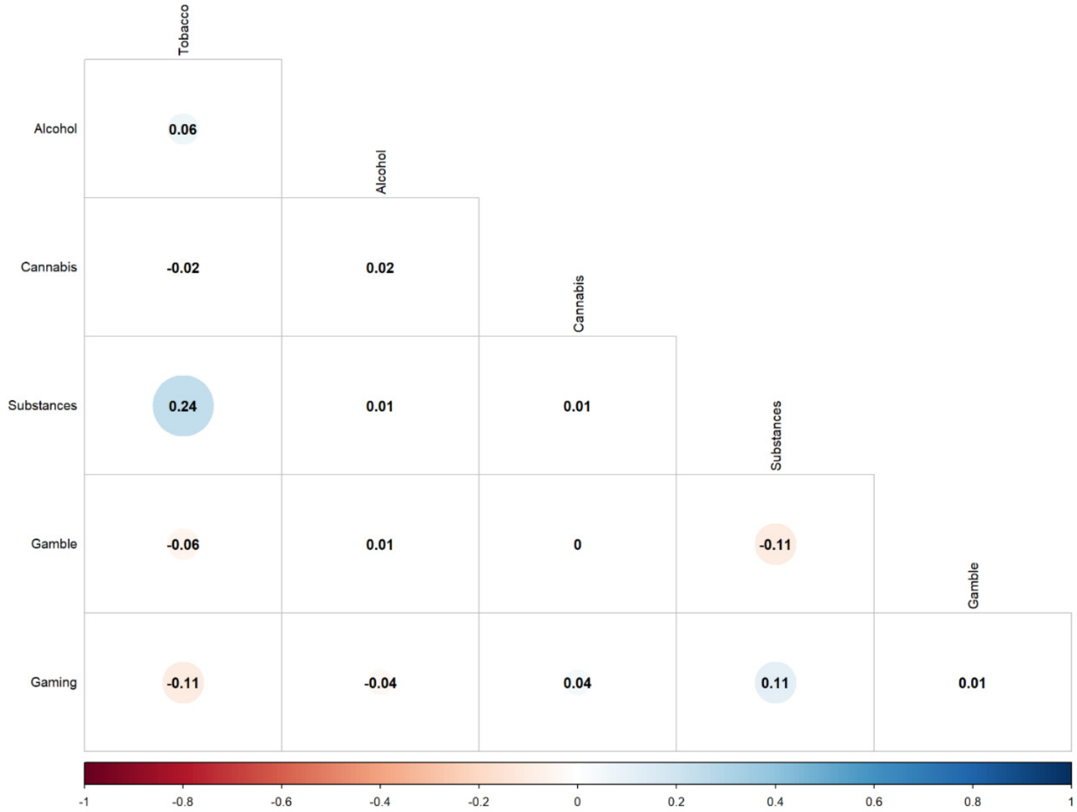


**Figure S9:** Weighted Pearson correlations table of the Gaming, Gamble, Substances, Cannabis, Alcohol and Tobacco. The size and the color scale of the circle in the correlation table, express the magnitude, and the sign of the correlation, respectively.

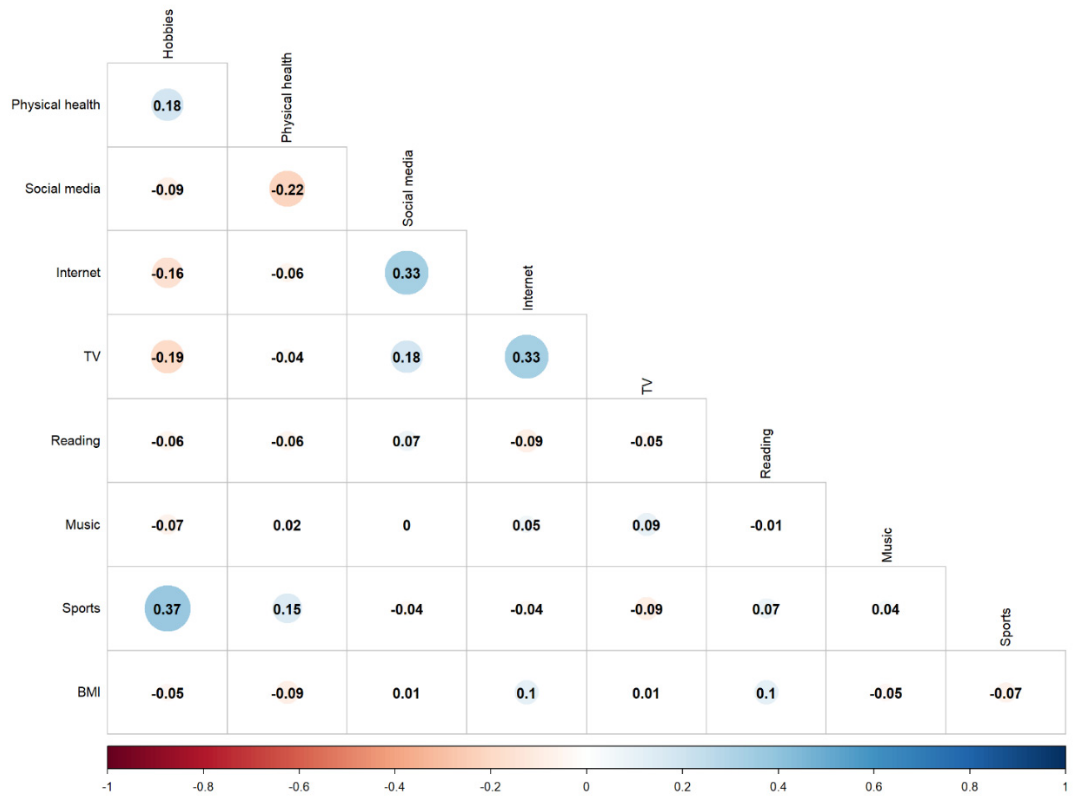


**Figure S10:** Weighted Pearson correlations table of the Hobbies, Physical health, Social media, Internet, TV, Reading, Music, Sports, and BMI. The size and the color scale of the circle in the correlation table, express the magnitude, and the sign of the correlation, respectively.

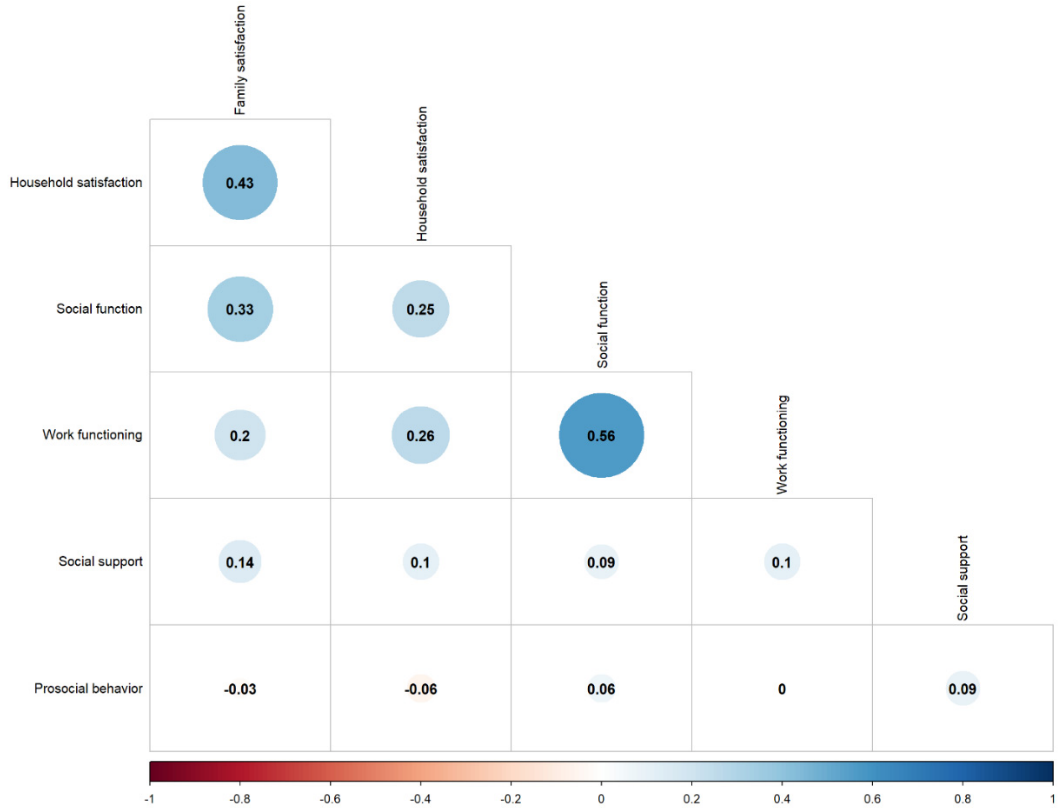


**Figure S11:** Weighted Pearson correlations table of the Family satisfaction, Household satisfaction, Social function, Work functioning, Social support, and Prosocial behavior. The size and the color scale of the circle in the correlation table, express the magnitude, and the sign of the correlation, respectively.
